# Metabolic, Mental and Immunological Effects of Normoxic and Hypoxic Training in Multiple Sclerosis Patients: A Pilot Study

**DOI:** 10.3389/fimmu.2018.02819

**Published:** 2018-11-29

**Authors:** Anja Mähler, Andras Balogh, Ilona Csizmadia, Lars Klug, Markus Kleinewietfeld, Jochen Steiniger, Urša Šušnjar, Dominik N. Müller, Michael Boschmann, Friedemann Paul

**Affiliations:** ^1^Experimental and Clinical Research Center Cooperation Between Charité Universitätsmedizin Berlin and Max Delbruck Center for Molecular Medicine, Berlin, Germany; ^2^Berlin Institute of Health, Berlin, Germany; ^3^German Centre for Cardiovascular Research Partner Site Berlin, Berlin, Germany; ^4^Max Delbruck Center for Molecular Medicine in the Helmholtz Association, Berlin, Germany; ^5^VIB Laboratory of Translational Immunomodulation, VIB Center for Inflammation Research Hasselt University, Diepenbeek, Belgium; ^6^International Centre for Genetic Engineering and Biotechnology, Triste, Italy; ^7^NeuroCure Clinical Research Center and Department of Neurology, Charité Universitätsmedizin Berlin Humboldt Universität Berlin, Berlin, Germany

**Keywords:** multiple sclerosis, hypoxia, endurance training, walking ability, energy metabolism, Tregs, Th17 cells

## Abstract

**Background:** Physical activity might attenuate inflammation and neurodegeneration in multiple sclerosis (MS). Erythropoietin, which is produced upon exposure to hypoxia, is thought to act as a neuroprotective agent in MS. Therefore, we studied the effects of intermittent hypoxic training on activity energy expenditure, maximal workload, serum erythropoietin, and immunophenotype focusing on regulatory and IL-17A-producing T cells.

**Methods:** We assigned 34 relapsing-remitting MS patients within a randomized, single blind, parallel-group study to either normoxic (NO) or hypoxic (HO) treadmill training, both 3 times/week for 1 h over 4 weeks (Clinicaltrials.gov identifier: NCT02509897). Before and after training, activity energy expenditure (metabolic chamber), maximal workload (incremental treadmill test), walking ability, depressive symptoms (Beck Depression Inventory I), serum erythropoietin concentrations, and immunophenotype of peripheral blood mononuclear cells (PBMCs) were assessed.

**Results:** Energy expenditure did not change due to training in both groups, but was rather fueled by fat than by carbohydrate oxidation after HO training (*P* = 0.002). Maximal workload increased by 40 Watt and 42 Watt in the NO and HO group, respectively (both *P* < 0.0001). Distance patients walked in 6 min increased by 25 m and 27 m in the NO and HO group, respectively (NO *P* = 0.02; HO *P* = 0.01). Beck Depression Inventory score markedly decreased in both groups (NO *P* = 0.03; HO *P* = 0.0003). NO training shifted Treg subpopulations by increasing and decreasing the frequency of CD39^+^ and CD31^+^ Tregs, respectively, and decreased IL-17A-producing CD4^+^ cells. HO training provoked none of these immunological changes. Erythropoietin concentrations were within normal range and did not significantly change in either group.

**Conclusion:** 4 weeks of moderate treadmill training had considerable effects on fitness level and mood in MS patients, both under normoxic and hypoxic conditions. Additionally, NO training improved Th17/Treg profile and HO training improved fatty acid oxidation during exercise. These effects could not be attributed to an increase of erythropoietin.

**Clinical Trial Registration:** ClinicalTrials.gov; NCT02509897; http://www.clinicaltrials.gov

## Introduction

Multiple sclerosis (MS) is an immune-mediated disease characterized by inflammation and neurodegeneration within the central nervous system (1). Physical activity might positively influence the inflammatory as well as the neurodegenerative aspects of the disease. Voluntary exercise in experimental autoimmune encephalomyelitis (EAE) mice attenuated disability, immune cell infiltration and preserved axons, and motor neurons in the spinal cord (2). Some evidence suggests a beneficial effect of exercise on cytokine responses and neurotrophic factors in MS patients (3–6). Cross-sectional studies have shown a positive association between cardiorespiratory fitness and gray matter volumes and white matter integrity in MS patients (7, 8).

Erythropoietin is thought to act as a neuroprotective agent in MS. Its application delayed disease onset, reduced inflammation and clinical scores in acute EAE in rats (9) and exerted positive effects in a small study on progressive MS patients (10) and as add-on therapy in acute optic neuritis (11). Serum erythropoietin naturally increases upon exposure to hypoxia. However, studies in healthy subjects, mostly athletes, showed a high inter-individual variability and no clear dose-response relationship has been established so far (12).

Training in hypoxia can induce similar training effects at a lower workload than training in normoxia (13), which could be beneficial in MS patients with reduced mobility. In a pilot study on the effects of intermittent hypoxia in MS patients, spasticity was significantly reduced after aerobic training under both normoxic and hypoxic conditions. However, only hypoxic training resulted in a significantly improved 6-min walk test, indicating improved endurance capacity (14).

Th1 and Th17 responses as well as malfunction of Tregs have been shown to contribute to the pathogenesis of MS by shifting the immune response toward inflammation (15–19). A limited number of small clinical studies showed that physical activity decreases IFN-γ and IL-17 plasma levels in MS patients (20, 21), implicating that physical activity could be beneficial for MS patients.

Therefore, we studied the effects of intermittent hypoxic training on serum erythropoietin, activity energy expenditure, maximal workload, and immunophenotype focusing on IFN-γ-, IL-17A-producing and regulatory T cells. We hypothesized that 4 weeks training under hypoxia would exert greater effects on efficiency of muscle work during moderate intensity exercise than training under normoxia.

## Methods

### Study Design

**Figure 1:**
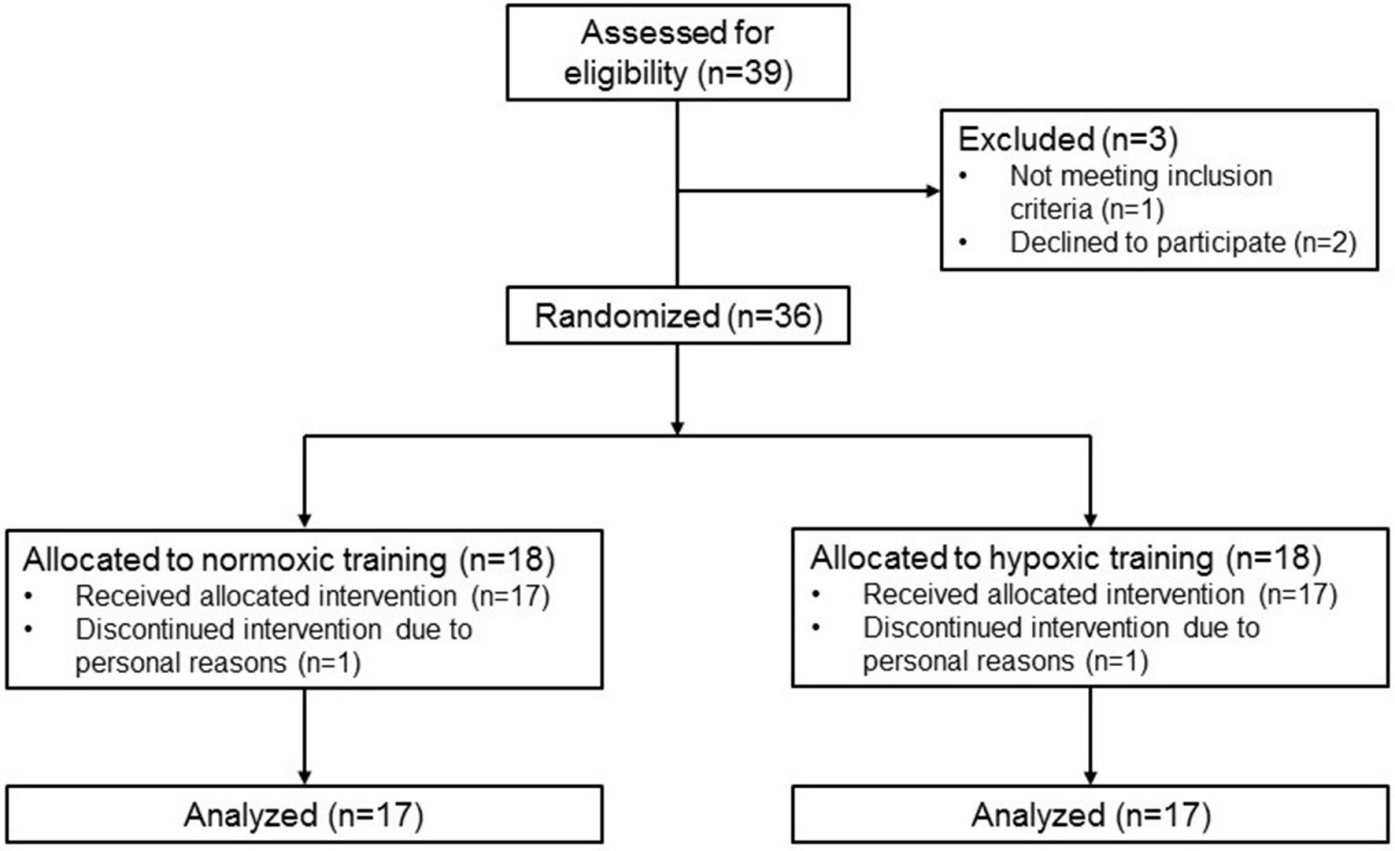
CONSORT 2010 flow diagram.

This was a randomized, single blind, controlled, parallel-group study conducted at the Experimental and Clinical Research Center of Charité Universitätsmedizin Berlin from July 2015 to April 2017 (ClinicalTrials.gov identifier: NCT02509897; Figure 1). Randomized assignment of patients to either normoxic or hypoxic training (1:1) was based on two computer-generated lists for single treatments, one for women and one for men. Patients and all investigators involved in immunological outcome measurements were blinded to the treatment allocation. However, persons who supervised the training sessions had to know the allocation in order to set the hypoxia chamber to the right condition. Training sessions were similar in both groups and moderate hypoxia was not perceivable by patients.

### Patients

We screened 39 patients with relapsing-remitting MS according to the 2010 panel criteria (22) from June 2015–March 2017 (last follow-up April 2017). Key inclusion criteria were a stable immune-modulatory therapy or no MS medication for at least 6 months, an expanded disability status scale (EDSS) score < 4.5 (23), age between 20 and 60 years, and a body mass index between 18.5 and 30.0 kg/m^2^. Key exclusion criteria were primary or secondary progressive forms of MS, clinical relapse within 3 months prior to or during the study, and clinically relevant heart, lung, liver, and kidney diseases. The institutional review board of Charité Universitätsmedizin Berlin approved the study. Written informed consent in accordance with the Declaration of Helsinki was obtained from all patients prior to study entry.

All patients lived in the greater Berlin area about 35 m above sea level and had not visited higher altitudes within the weeks before the study. We advised patients to continue their habitual activity level and lifestyle throughout the study.

### Hypoxia Chamber

Training sessions were conducted in a normobaric hypoxia chamber (11 m^2^, 38 m^3^; Linde AG, Berlin, Germany). Oxygen content within the room was reduced by mixing the incoming air with nitrogen delivered from a nitrogen tank containing liquid nitrogen. Oxygen and carbon dioxide concentrations within the room were continuously monitored throughout the training sessions with two redundant sensors for each gas and the inflow of fresh air and nitrogen were automatically adjusted accordingly.

### Training Intervention

Patients were submitted to a 4-week training program. During training, they briskly walked on a motorized treadmill (h/p/cosmos mercury 4.0, h/p/cosmos sports and medical GmbH, Germany) at 65% of their individual maximal heart rate. One group trained under normobaric normoxia (21% FiO_2_, NO), the other one under normobaric hypoxia corresponding to 2,500 m altitude (15% FiO_2_, HO). Individual maximal heart rate was determined beforehand in an incremental treadmill test. All patients attended 12 training sessions within 4 weeks. Each session lasted 1 h, during which patients walked three times 15 min on the treadmill followed by a 5 min break. Heart rate was monitored continuously and either pace or slope of the treadmill was adjusted by the supervisor to maintain the required heart rate.

### Study Protocol

Before (V1) and after training (V2), patients were tested after a 12-h overnight fast. Additionally, all patients were asked to refrain from caffeine and alcohol containing beverages on the preceding day and from smoking on the study day itself. We obtained venous blood for erythropoietin and blood count measurements and for peripheral blood mononuclear cell (PBMC) isolation. Body composition was determined by Air-Displacement Plethysmography (Bod Pod, Life Measurements, Inc., Concord, CA). Carbon dioxide production (VCO_2_) and oxygen consumption (VO_2_) were measured in a metabolic chamber - a comfortable, airtight room (5 m^2^, 11 m^3^) that is constantly supplied with fresh air like an open circuit indirect calorimeter (24). VO_2_ and VCO_2_ were used to assess changes in energy expenditure (EE) and respiratory exchange ratio (RER = VCO_2_/VO_2_).

While being seated in a comfortable chair, resting EE was measured over 40 min followed by a 75 g oral glucose load. Then, patients started exercising on a bicycle ergometer (VIAsprint 150 P, Ergoline, Germany) at a workload of 0.5 W/kg body weight over 40 min. During exercise, heart rate was monitored continuously and rates of perceived exertion on a 10-point scale (25) were recorded every 10 min. Exercise was followed by a recovery period (40 min).

After EE measurements, patients had a 60 min break for taking breakfast. Then, they underwent a 10 m walk test (10 mWT) and a 6 min walk test (6 MWT). For the 10 mWT, patients were asked to walk a 10 m long course as quickly as possible. The test was repeated three times and the mean gait speed was used for assessment. For the 6 MWT, patients were asked to walk a 25 m long course up and down for 6 min while the covered distance was measured. The 10 mWT is a validated measure of walking ability, whereas the 6 MWT rather measures walking endurance (26). After another break, patients completed an incremental VO_2max_ test on a motorized treadmill. The test was adapted from Langeskov-Christensen et al. who tested the validity and reliability of VO_2max_ measurements in MS patients vs. healthy controls (27). We decided to use a treadmill protocol because walking is more important for daily life activities than cycling. The test started with a 5 min warm-up at a self-chosen pace between 3.5 and 5.3 km/h. Then the treadmill was switched to a steeper slope every 1.5 min until exhaustion. Increments were calculated individually based on sex, age, height and weight in order to exhaust patients within 10 min (28). During the test, breath-by-breath gas exchange and an electrocardiogram were recorded (Quark, COSMED, Italy). Before the test and within one min after exhaustion, blood was obtained from an earlobe to determine blood lactate concentrations.

### Questionnaires

Possible changes in fatigue and self-reported depressive symptoms were evaluated with the Fatigue Severity Scale (FSS), the Modified Fatigue Impact Scale (MFIS), and the Beck Depression Inventory (BDI-I), respectively. All three instruments are commonly used and validated in MS patients (29–31).

The habitual physical activity level of patients was evaluated with a questionnaire asking for work, leisure, and sports activities within the preceding year (32). Resting EE per hour was calculated from the baseline EE measurement in the metabolic chamber and the respective values were multiplied by energy cost and duration (hour/week) of the respective activity (33).

### Peripheral Blood Mononuclear Cell Analysis

PBMCs were processed and analyzed as described previously (34). Briefly, peripheral venous blood was obtained and mononuclear cells were isolated within 4 h by density gradient centrifugation using Biocoll (Merck, Darmstadt, Germany) and cryopreserved until further processing. Thawed cell aliquots were either labeled for extracellular antigens using fluorophore-conjugated monoclonal antibodies or CD4^+^ cells were selected (Miltenyi CD4^+^ Selection Kit). Cells (10^6^) from CD4^+^ and CD4^−^ fractions were placed onto U-bottom plates and re-stimulated for 4 h at 37°C and 5% CO_2_ in a humidified incubator in a final volume of 200 μl RPMI 1640 (Sigma) supplemented with 10% FBS (Merck), 100 U/ml penicillin (Sigma), 100 mg/ml streptomycin (Sigma), 50 ng/ml phorbol 12-myristate 13-acetate (Sigma), 250 ng/ml ionomycin (Sigma), and 1.3 μl/ml Golgistop (BD). After re-stimulation, cells were labeled with Aqua Life/Dead Viability Staining kit (Invitrogen) for discrimination between dead and viable cells. Furthermore, cells were labeled with respective surface antigen-specific fluorophore-conjugated monoclonal antibodies, then fixated and permeabilized by FoxP3/Transcription Factor Staining Kit (eBioscience), and subsequently labeled with respective intracellular-antigen-specific fluorophore-conjugated monoclonal antibodies. Samples were analyzed using FACSCanto II multicolor flow cytometer (BD). Gating strategy of the flow cytometry analysis is shown in Figure S1. Data analysis was performed with FlowJo 10.3 (FlowJo LLC) and FCSExpress V6.02 (De Novo Software) software.

### Outcome Measures

Primary outcome measure was an improved efficiency of muscle work during moderate intensity exercise assessed by indirect calorimetry (metabolic chamber) after 4 weeks training under hypoxia vs. normoxia.

Secondary outcome measures were maximal workload (incremental treadmill test), walking ability (10 mWT and 6 MWT), serum erythropoietin concentrations and immune-regulation of lymphocytes (Th17 cell/Treg balance), all after 4 weeks training under hypoxia vs. normoxia.

### Statistical Analysis

This was a pilot study. Thus, all tests should be understood as constituting exploratory data analysis. Therefore, no sample size calculation or adjustments for multiple testing were made. Statistical analyses were performed with GraphPad Prism (versions 6.01 and 7.01) and Charles Zaiontz Real Statistics Resource Pack software (Release 5.4). Data in graphics are shown as single values or as mean and standard error of the mean. For clinical data, two-way ANOVA followed by Sidak *post-hoc* test was used and the *P-*values for training effects within one group (before vs. after training) and differential training effects between groups (NO vs. HO) are shown in the figures.

For immunological data, distribution of samples was tested by Shapiro-Wilk test using Real Statistics Resource Pack. Depending on their distribution, training effects within one group (before vs. after training) were compared by Student's two two-tailed *t-*test or non-parametric Wilcoxon's test. Training effects between groups (NO vs. HO) were compared by Student's unpaired two-tailed *t-*test or Mann-Whitney U test. A *P-*value < 0.05 indicated statistical significance.

## Results

Demographic and anthropometric characteristics of the 34 MS patients (all Caucasians) who completed the study are summarized in Table 1. There were no clinically relevant differences in the baseline characteristics of the NO and HO group. Disease modifying therapies were diverse among patients and distribution was comparable between groups (Table 1).

**Table 1:** Baseline characteristics of 34 MS patients^1^.

	**Normoxia**	**Hypoxia**
Women/men	11/6	11/6
Age, years	51 (10)	49 (9)
BMI, kg/m^2^	24.0 (4.4)	25.3 (4.8)
Body fat, %	29.1 (10.1)	29.5 (10.2)
PAL	1.72 (0.27)	1.76 (0.27)
Disease duration, months^2^	158 (35–456)	156(2–336)
EDSS, arbitrary units^2^	3.0 (0.0–4.0)	3.0 (1.0–4.0)
No immunomodulatory therapy (*n*)	4	4
Immunomodulatory therapy (*n*)	13	13
Interferons (*n*)	3	6
Glatiramer acetate (*n*)	3	2
Dimethyl fumarate (*n*)	4	3
Teriflunomide (*n*)	3	1

1 Data are given as means (SD) unless stated otherwise. BMI, body mass index; PAL, physical activity level; EDSS, expanded disability status scale.

2 *Median (total range)*.

### Systemic Energy Metabolism

**Figure 2:**
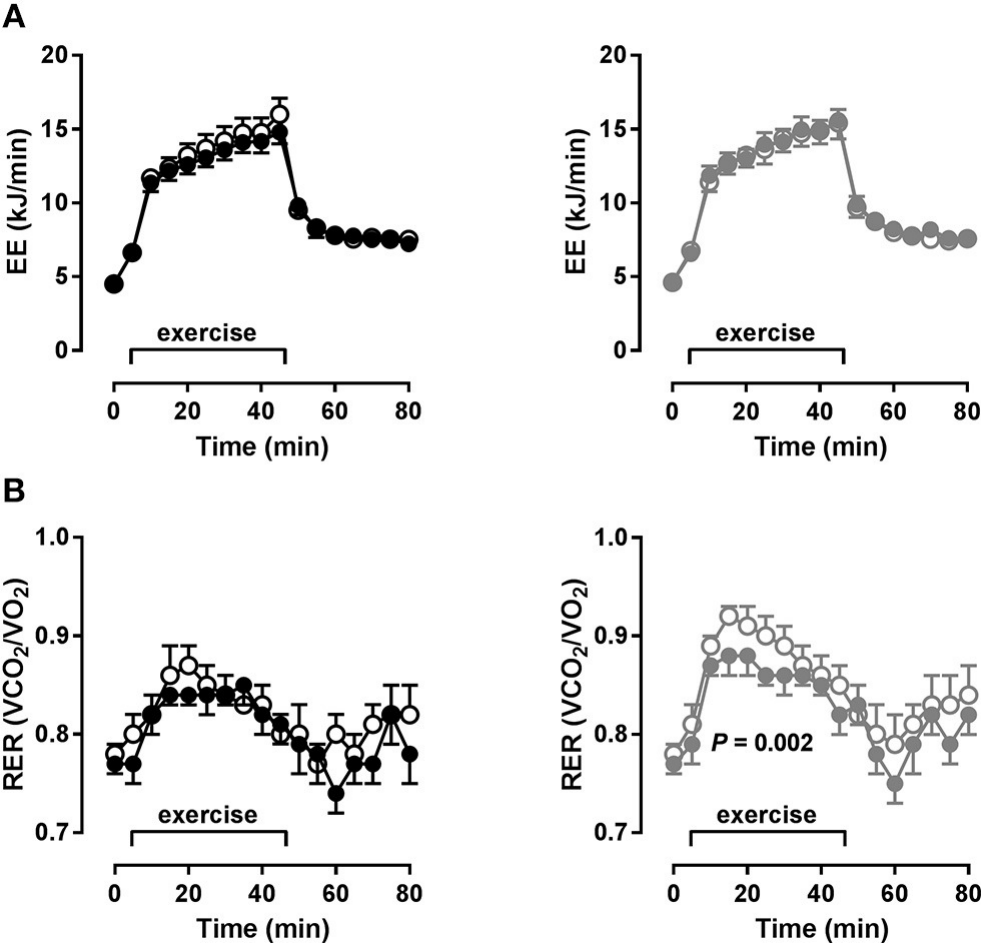
Systemic energy metabolism during exercise (metabolic chamber). **(A)** Energy expenditure (EE) and **(B)** respiratory exchange ratio (RER) at rest and during bicycle exercise after glucose in MS patients before (open circles) and after (closed circles) 4 weeks of normoxic (NO, *n* = 16, black circles) and hypoxic (HO, *n* = 17, gray circles) treadmill training. Data as mean (SEM), *P*-value (training) by ANOVA.

We investigated systemic metabolism at rest and during moderate bicycle exercise. Baseline resting EE did not differ significantly between the groups and did not change due to training in either group (Figure 2A). After starting exercise, EE increased immediately within the first 10 min and more slowly during the following 30 min without reaching a steady state in both groups. Total increase in EE within 40 min exercise did not differ between the groups, indicating comparable fitness levels at baseline. During the recovery period, EE decreased regularly but did not reach resting levels in both groups. Neither NO nor HO training had significant effects on EE during moderate bicycle exercise (Figure 2A).

Baseline resting RER did not differ between the groups (NO: 0.78 ± 0.03; HO: 0.78 ± 0.04), again indicating well matched groups, also on a metabolic level. After 4 weeks of training, resting RER were virtually the same (NO: 0.77 ± 0.04; HO: 0.77 ± 0.04). In the NO group, RER increased to a lesser extent during exercise at baseline and there was only a trend toward decreased RER during exercise after the training (*P* = 0.07). This indicates an already sufficient fatty acid oxidation at baseline with only slight improvement due to training. However, in the HO group, RER increased more potently at baseline and was significantly lower after training (*P* = 0.002), indicating an improved fat oxidation due to training (Figure 2B).

### Primary Outcome Measure

**Figure 3:**
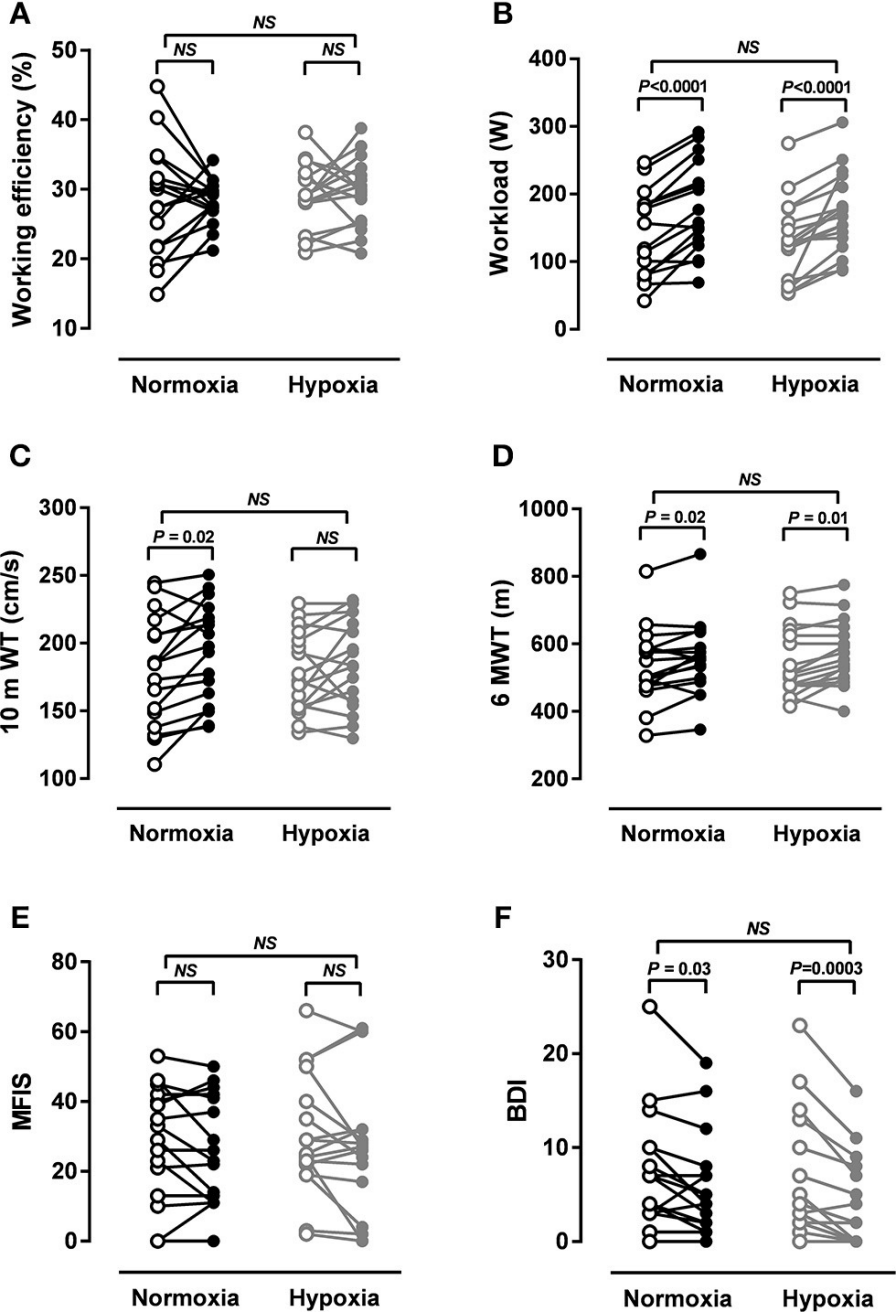
Fitness, walking ability, fatigue and depression. **(A)** Working efficiency, **(B)** maximal workload in an incremental treadmill test, **(C)** gait velocity in a 10 m walking test (10 mWT), **(D)** walking endurance in a 6 min walking test (6MWT), **(E)** Modified Fatigue Impact Scale (MFIS), **(F)** Beck Depression Inventory (BDI-I). All in MS patients after 4 weeks of normoxic (NO, *n* = 16, black circles) and hypoxic (HO, *n* = 17, gray circles) treadmill training. *P-*values by ANOVA and Sidak *post-hoc* test.

Energy efficiency (workload/EE during exercise) did not change in either group due to training (NO: 28.4 ± 8.1% vs. 28.3 ± 3.2%, *NS*; HO: 28.9 ± 4.8% vs. 29.5 ± 5.0%, *NS;* Figure 3A). Although energy efficiency, i.e., efficiency of muscle work during bicycle exercise, was rather unchanged, other relevant secondary measures of performance did improve.

### Physical and Mental Effects Of Training

At baseline, VO_2max_ in an incremental treadmill test was 32.8 ± 7.5 ml/min/kg BW in the NO group and 30.3 ± 7.1 ml/min/kg BW in the HO group. VO_2max_ did not change markedly due to training in either group (NO: 33.4 ± 7.6 ml/min/kg BW, HO: 30.5 ± 7.7 ml/min/kg BW; both *NS*). At baseline, maximal workload was 141 ± 64 W in the NO group and 130 ± 60 W in the HO group. After training, workload was increased by 40 W in the NO group (*P* < 0.0001) and by 42 W (*P* < 0.0001) in the HO group (Figure 3B). HO training was not superior to NO training in improving maximal workload.

At baseline, gait speed was 179 ± 41 cm/s in the NO group and 178 ± 30 cm/s in the HO group. Gait speed significantly increased in the NO group (194 ± 37 cm/s, *P* = 0.02) and slightly increased in the HO group (185 ± 33, *NS*). Although the improvement of 8% in the NO group is not considered clinically meaningful, it is noteworthy that especially the slowest patients in this group improved whereas those with almost normal gait speed did not change (Figure 3C). At baseline, distances patients walked in 6 min were 534 ± 110 m in the NO group and 549 ± 102 m in the HO group. Distances increased by 25 m in the NO group (*P* = 0.02) and by 27 m in the HO group (*P* = 0.01; Figure 3D). HO training was not superior to NO training in improving gait parameters.

Baseline FSS was 3.6 ± 1.6 in the NO group and 3.7 ± 1.7 in the HO group. Thus, mean values of the groups did not indicate fatigue. However, seven patients in each group had scores between 4.0 and 6.4 before the training. Although mean severity of fatigue did not change in either group, 50% of fatigued patients had lower scores after the training (two after NO and five after HO training). Baseline MFIS was 26.8 ± 17.2 in the NO group and 30.2 ± 17.2 in the HO group. In line with the severity of fatigue, its impact on daily activities did not change in either group (Figure 3E). Baseline BDI scores were 7.5 ± 6.1 in the NO group and 7.1 ± 6.6 in the HO group. BDI scores significantly decreased in both groups (NO−2 points, *P* = 0.03; HO−3 points, *P* = 0.0003). Although baseline means did not indicate depression, five and six patients in the NO and HO group, respectively, were classified as depressed (values > 10). Two and four of them were below 10 after NO and HO training, respectively (Figure 3F). HO training was not superior to NO training in changing fatigue or depression.

Surprisingly, none of these changes were mediated through changes in EPO. Concentrations were within normal range at baseline and did not significantly change due to training in either group (NO: 9.4 ± 3.2 vs. 10.6 ± 3.5 mIU/ml, *NS*; HO: 8.6 ± 4.1 vs. 9.0 ± 3.7 mIU/ml, *NS*). Also other markers of erythropoiesis like reticulocyte and red blood cell count did not change due to training (data not shown).

Venous glucose and triglyceride concentrations were within normal range at baseline and did not significantly change due to training in either group. However, after training HDL cholesterol was significantly higher in the NO group and total cholesterol and LDL cholesterol were significantly higher in the HO group (Table 2).

**Table 2:** Serum metabolic markers of MS patients before and after 4 weeks of normoxic (*n* = 17) and hypoxic treadmill training (*n* = 17).

	**Normoxia**		**Hypoxia**	
	**Before**	**After**	**Before**	**After**
Glucose, mg/dl	89 (10)	87 (13)	88 (8)	84 (12)
Total cholesterol, mg/dl	228 (51)	237 (40)	207 (46)	221 (50)^*^
LDL cholesterol, mg/dl	140 (48)	142 (36)	124 (42)	134 (45)^*^
HDL cholesterol, mg/dl	68 (16)	72 (18)^*^	64 (19)	67 (18)
Total/HDL ratio	3.5 (1.1)	3.5 (1.1)	3.4 (1.0)	3.5 (1.0)
LDL/HDL ratio	2.2 (0.9)	2.1 (0.9)	2.1 (0.9)	2.2 (0.9)
Triglycerides, mg/dl	104 (67)	114 (68)	95 (35)	97 (35)

Data are given as means (SD). LDL, low density lipoprotein; HDL, high density lipoprotein.

* *P < 0.05 before vs. after training (Wilcoxon signed rank test)*.

### Immunological Effects of Training

**Figure 4:**
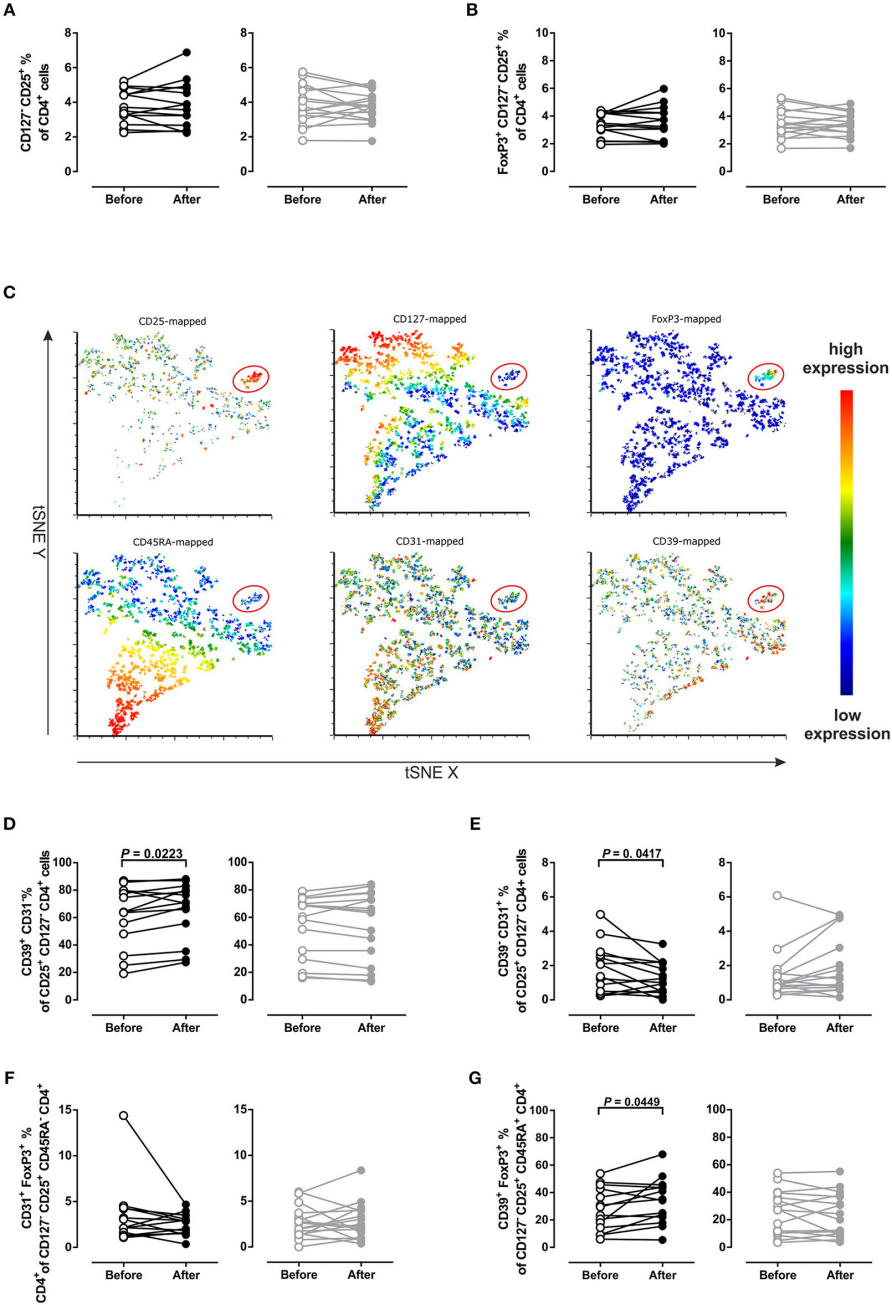
Frequency changes of different regulatory T cell populations. **(A)** Regulatory T cells defined as CD4^+^ CD25^+^ CD127^−^ or **(B)** FoxP3^+^ CD4^+^ CD25^+^ CD127^−^. **(C)** t-Distributed Stochastic Neighbor Embedding (t-SNE) plot for the expression-distribution of CD25, CD127, FoxP3, CD45RA, CD31 and CD39 within one representative sample (red ellipses indicate regulatory T cells). **(D)** CD39^+^ CD31^−^ and **(E)** CD39^−^ CD31^+^ Tregs defined as being CD25^+^ CD127^−^ CD4^+^. **(F)** Memory phenotype CD31^+^ FoxP3^+^ CD45RA^−^ Tregs and **(G)** naïve phenotype CD39^+^ FoxP3^+^ CD45RA^+^ Tregs in MS patients after 4 weeks of normoxic (NO, *n* = 16, black circles) and hypoxic (HO, *n* = 14, gray circles) treadmill training. Three samples not analyzed due to quality issues. *P-*values by Student's paired *t-*test or Wilcoxon signed rank test.

We performed an intensive immunophenotyping of our patients. Using flow cytometry, we focused on CD4^+^ regulatory T cell (Treg) and CD4^+^ T helper cell (Th) populations. Frequency of Tregs characterized either as CD4^+^ CD127^−^ CD25^+^ or, more specifically, as CD4^+^ CD127^−^ CD25^+^ expressing the Treg-specific transcription factor FoxP3^+^ (referred throughout this paper as FoxP3^+^ Tregs) did not change due to training in either group (Figures 4A,B, Figure S2A).

However, additional analysis of the expression patterns by t-Distributed Stochastic Neighbor Embedding (t-SNE) analysis, which takes the expression of the CD markers CD31 (PECAM-1), CD39 (ectonucleoside triphosphate diphosphohydrolase-1^+^, NTPDase1^+^) and CD45RA into account, showed that the FoxP3^+^ Treg population was heterogeneous for the above markers (Figure 4C). This led us to analyze other distinct populations of Tregs, such as CD45RA^−^ CD31^+^ FoxP3^+^ and CD45RA^+^ CD39^+^ FoxP3^+^ Tregs (Figures 4F,G). This analysis revealed that NO training increased the frequency of CD39^+^ CD31^−^ Tregs (*P* = 0.02; Figure 4D) and CD39^+^ Tregs, *P* = 0.03; Figure S2B). Further, frequency of CD39^−^ CD31^+^ Tregs was decreased after NO training (*P* = 0.04; Figure 4E, Figure S2C).

Frequency of CD45RA^−^ CD31^+^ FoxP3^+^ Tregs did not change (Figure 4F), whereas CD45RA^+^ CD39^+^ FoxP3^+^ Tregs were higher after NO training (*P* = 0.05; Figure 4G). Counterpart proportions of these Treg populations did not change due to training (CD39+ CD45RA- FoxP3+, CD31+ FoxP3+ CD45RA+, Figure S2D,E, respectively).

**Figure 5:**
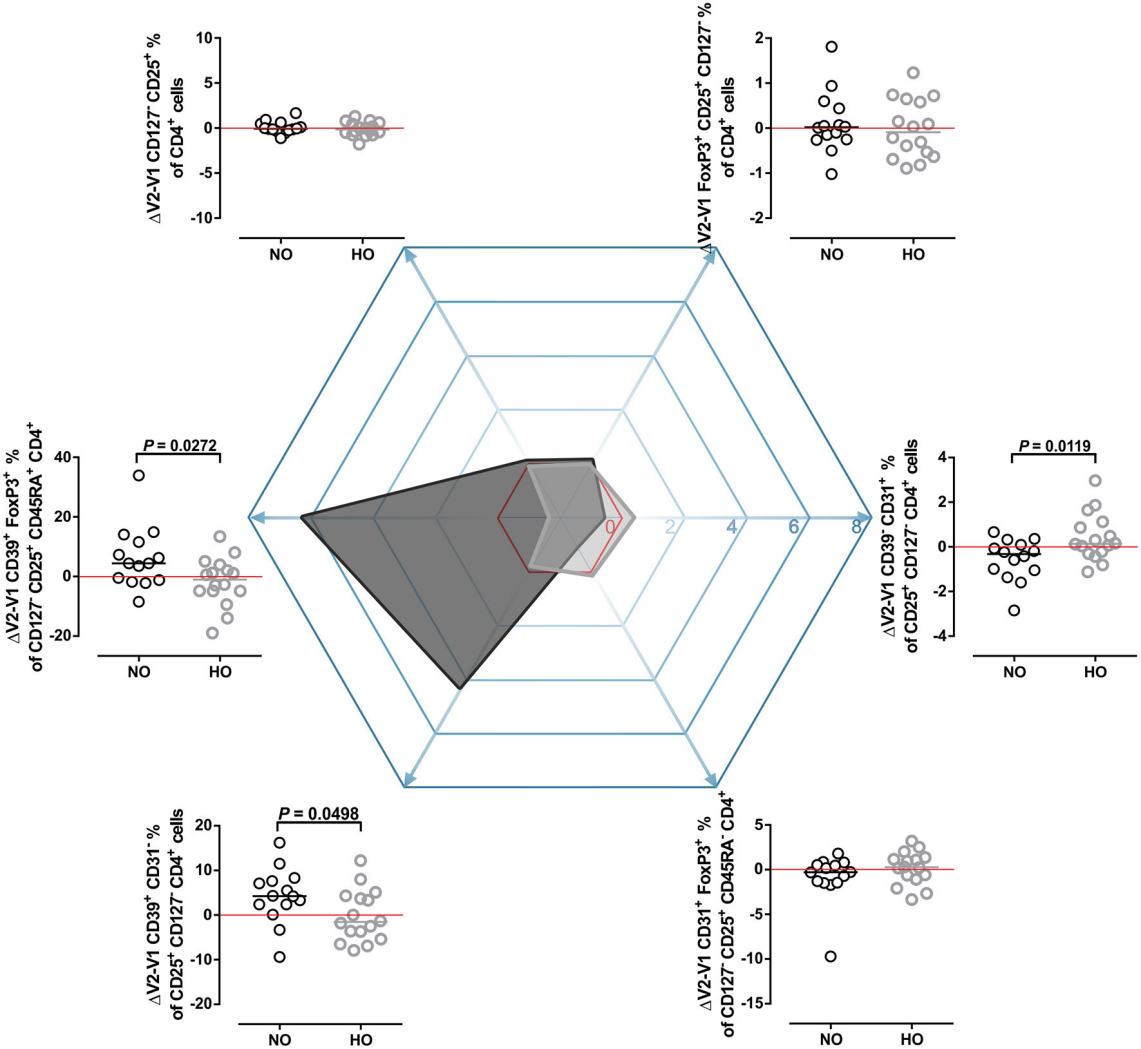
Shifts toward CD39^+^ and CD31^+^ Treg phenotypes. Differences between V2 and V1 (ΔV2-V1) of the examined Treg populations were compared and are shown around a hexagon radar chart. ΔV2-V1 percentage point values were plotted on the matching axis of the radar chart. Positive values indicate an increase, negative values indicate a decrease of the measured parameter after training. Red lines (no change), NO (black circles and dark gray shaded area on radar chart), HO (gray circles and light gray shaded area on radar chart). *P-*values by Student's *t-*test or Mann-Whitney *U-*test.

To compare training effects, we assessed differences of Treg populations between V2 and V1 (ΔV2-V1; Figure 5). NO and HO training differentially changed CD39^−^ CD31^+^ (*P* = 0.01), CD39^+^ CD31^−^ Tregs (*P* = 0.05) and CD39^+^ FoxP3^+^ CD45RA^+^ Tregs (*P* = 0.03). However, at the level of Tregs training effects were not different (Figure 5, Figure S2A). In the NO group, ΔV2-V1 values revealed a clear shift toward the CD39^+^ direction with a subsequent decrease of CD31^+^ positivity. In contrast, HO training promoted a shift toward the CD31^+^ Treg direction with a consequent decrease of the CD39^+^ phenotype (Figure 5).

**Figure 6:**
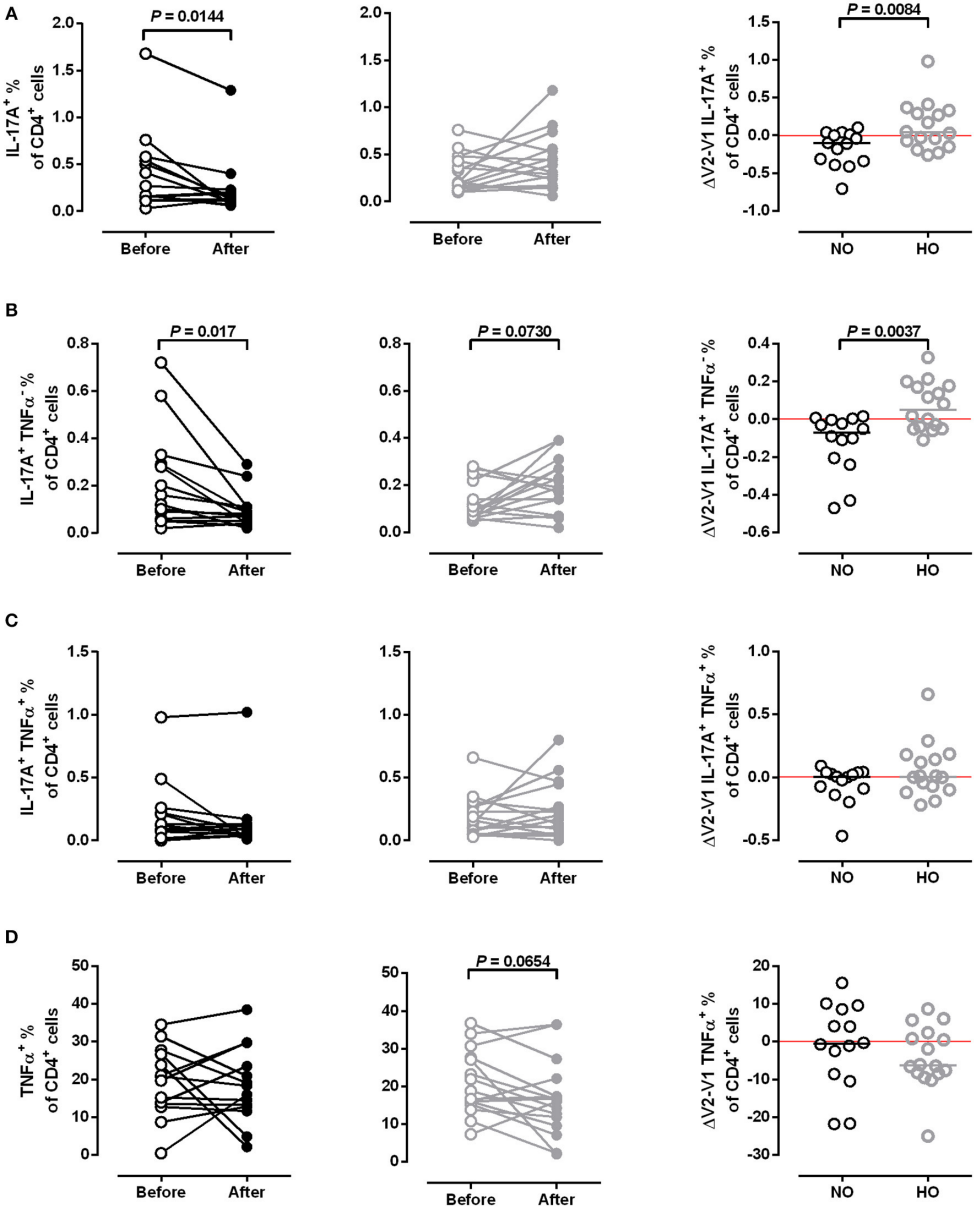
Frequency of IL-17A and TNFα producing T cells. Frequencies of **(A)** total IL-17A^+^, **(B)** IL-17A^+^ TNFα^−^, **(C)** IL-17A^+^ TNFα^+^, and **(D)** TNFα^+^ CD4^+^ cells in MS patients after 4 weeks of normoxic (NO, *n* = 16, black circles) and hypoxic (HO, *n* = 14, gray circles) treadmill training. Three samples not analyzed due to quality issues. Comparison of training effects (ΔV2-V1, right column). *P*-values before vs. after training by Student's paired *t-*test or Wilcoxon signed rank test. *P-*values NO vs. HO by Mann-Whitney *U* test or Student's *t-*test.

Furthermore, we evaluated pro inflammatory cytokines such as IL-17A, TNFα and IFN-γ. For this, CD4^+^-enriched cell fractions were *in vitro* re-stimulated in order to investigate cytokine expression of CD4^+^ T cells by flow cytometry. NO training significantly decreased the frequency of total IL-17A^+^ (*P* = 0.01) and IL-17A^+^ TNFα^−^ (*P* = 0.02) CD4^+^ cells (Figures 6A,B), but did not affect the frequency of IL-17A^+^ TNFα^+^ and total TNFα^+^ CD4^+^ cells (Figures 6C,D). HO training did not change any of these CD4^+^ cell populations.

Neither NO nor HO training altered frequencies of IFN-γ^+^, IFN-γ^+^ IL-17A^+^, IFN-γ^+^ IL-17A^−^ CD4^+^ T cells (Figure S3). Furthermore, median fluorescence intensity (MFI) of IFN-γ-labeling of CD4^+^ cells showed that IFN-γ-MFI of these cells was not altered after both trainings (Figure S4).

## Discussion

Physical activity is recommended as an important complementary tool in MS therapy (35, 36). Here we studied metabolic, mental and immunological effects of a moderate treadmill training and hypothesized that intermittent hypoxic training would add to the beneficial effects of a normoxic training. We investigated 34 patients with relapsing remitting MS who were comparable for age, body mass index, body fat content, physical activity level, disease duration, and EDSS. The men to women ratio was about 1:2. Since both groups were well matched, differential intervention effects are less likely to be attributable to any of the baseline characteristics.

Primary outcome measure was an improved energy efficiency during moderate intensity exercise. Expanding less energy for the same intensity of exercise would point to an improved fitness level. Energy efficiency of patients in this study was comparable with that of healthy persons (27.6 ± 4.5%; own unpublished data). Four weeks of moderate treadmill training did not increase energy efficiency during bicycle exercise. However, extreme baseline values in the NO group were within normal range after training. Energy efficiency might have increased with longer training duration. Also, due to shortage of space in the metabolic chamber, patients were tested on a bicycle instead of a treadmill.

Despite unchanged energy expenditure, HO training markedly increased fatty acid oxidation during exercise. This is in line with two other studies which found a shift toward fatty acid oxidation in healthy men after exercising in hypoxia (37) and in overweight men resting in hypoxia (38). In a previous study, we found increased carbohydrate and therefore decreased fatty acid oxidation during exercise in MS patients compared to healthy controls (24). Improved fatty acid oxidation, and thereby preservation of carbohydrate stores, might improve management of energy resources in MS patients. Thus, other relevant training effects observed after hypoxic training might be due to improved energy metabolism.

A meta-analysis of 40 studies showed that weight-adjusted VO_2max_ values of MS patients were significantly lower compared to healthy controls, and that aerobic training could increase VO_2max_ by 3.5 ml/min/kg BW (39). However, baseline VO_2max_ values of our patients were comparable to those of healthy individuals. Neither training increased VO_2max_, presumably due to insufficient training time and intensity. However, maximal workload increased by about 40 W or 30% in both groups. This increase is much higher than that attained in other MS studies, where increases ranged from 5 to 12 W or 4 to 11% (40–42). Thus, a 4-week treadmill training seems to be more effective for increasing maximal workload than bicycle training applied in most studies. However, familiarization with walking on a treadmill could have contributed to these higher increases.

Walking is frequently affected in MS patients, which negatively impacts on activities of daily life and contributes to decreased quality of life (26, 43). Moreover, gait function is perceived as the most valuable bodily function by MS patients (44) and is, therefore, one of the most relevant objectives in MS care. Gait speed was improved only after NO training, although the change was less than 20% and, therefore, not considered to be clinically meaningful (26). However, it is noteworthy that especially the slowest patients in this group improved. Thus, for those most impaired, training made a relevant difference. Mean distances patients walked in 6 min before training were about 90 m shorter than those of healthy subjects aged 50–85 years (45). Training markedly increased walking distances by about 25 m in both groups. Our patients recognized this improvement as the most valuable outcome of the study (oral communication).

Although exercise seems to be moderately effective in the treatment of fatigue in MS patients, effects are variable between studies and subjects (46). We found no improvement of fatigue, possibly because 65% of our patients were considered as non-fatigued with FSS scores below 4.6 and MFIS scores below 38 (47). Depression is two to five times more common in MS patients than in the general population, with 25–50% of patients developing major depression (48). Although our patients had only minimal, mild or moderate depressive symptoms, we found a marked decrease of BDI scores in both groups. This is in line with Rasova et al. who found decreased scores after physiotherapy, aerobic training and a combined program (49). Although it is not clear if depression is always secondary to immunological changes in MS (48), immunological effects observed in our study could have decreased depressive symptoms, especially after NO training.

A total of 12 h exposure to normobaric hypoxia corresponding to 2,500 m altitude over 4 weeks was obviously not sufficient to induce an increased EPO production. There is little data on the time course of EPO production in humans. One small study showed that continuous exposure to normobaric hypoxia corresponding to 5,500 m for 120 min sufficiently increased EPO production (50). However, both this altitude and intervention time would not have been feasible in our training study with MS patients due to safety issues and time constraints of participants. Nevertheless, intermittent normobaric hypoxia might result in EPO-dependent and EPO-independent effects.

Physical activity can improve blood lipid profile, which is commonly affected in MS. At baseline, total and LDL cholesterol levels were borderline high in both groups and increased slightly and markedly in the NO and HO group, respectively. However, HDL cholesterol levels were above 60 mg/dl in both groups both before and after the training, indicating protection against heart disease. Slightly increased total and HDL cholesterol were also reported in some case-control studies in MS (51). Total/HDL and LDL/HDL cholesterol ratios were about 3.5 and 2 in both groups, respectively, indicating a low atherogenic risk.

Besides physical and mental health, immunological status is important for disease progression in MS. MS is a chronic autoimmune disease, previously considered to be predominantly a disease with a Th1 skew, yet, there is a growing body of evidence showing the role of Th17 answer in the pathogenesis of MS (19). Besides the Th1-Th17 skew, genetic predisposition, CD8^+^ cytotoxic T cells, low levels of vitamin D (52, 53) and malfunctioning Treg homeostasis have been shown to contribute to the pathology of the disease (17).

Physical activity has been shown to affect immune responses in athletes, healthy volunteers and MS patients (21, 54–57). In a murine asthma model and in elite swimmers, exercise increased the frequency of Tregs (56, 58). In our study, neither NO nor HO training had a significant effect on the frequency of Treg cells. There were, however, trends for an increase after NO and a decrease after HO training.

Treg malfunction plays a role in the pathogenesis of MS (17). Naïve Treg function seems to be disturbed in early and late stages of MS, whereas memory Treg homeostasis recovers in the progressive phase (16, 17). CD39^+^ and CD31^+^ Tregs represent two different modalities. CD39 expression has been previously measured predominantly in FoxP3^+^ CD45RO^+^ T cells (59, 60), which possess a highly suppressive and anti-inflammatory capacity by removing free ADP/ATP (60). Additionally it was demonstrated that the frequency of circulating CD39^+^ Tregs is decreased in relapsing-remitting MS patients compared to healthy volunteers (60). Furthermore, Fletcher et al. showed that CD39^+^ CD25^high^ CD4^+^ Tregs suppress IL-17 production, while their CD39^−^ CD25^high^ CD4^+^ counterparts could produce IL-17 (18). Conversely, previous reports indicate that the adhesion molecule CD31 is expressed mostly on CD45RA^+^ recent thymic emigrant Tregs (59, 61, 62). In line with previously published data, we confirmed this distribution. However, and of interest, in the present study a notable number of CD45RA^+^ Tregs were CD39^+^ positive, and several CD45RA^−^ Tregs were capable of expressing CD31. Thus, it would be tempting to speculate that the training regimen could have effects on functionality of Treg subpopulations. However, this needs to be addressed in future studies. NO training had a beneficial effect on the immunophenotype by promoting CD45RA^+^ CD39^+^ CD31^−^ Tregs and decreasing their CD45RA^−^ CD39^−^ CD31^+^ counterparts shifting the Tregs toward CD39 phenotype. However, HO training did not exert this effect.

There are only a few studies investigating the effect of training on IL-17-producing cells. Combined exercise training (24 sessions over 8 weeks) decreased IL-17 and IFN-γ in plasma and supernatants of cultured PBMCs from women with relapsing-remitting MS (20). In line with this, cultured PBMCs from patients with relapsing-remitting MS, who had trained 2 h per week for 12 weeks, produced significantly less IL-17 (21). We applied flow cytometry to investigate the IL-17A and TNFα-expression of CD4^+^ cells, which are considered to be the major source of IL-17A. The frequency of IL17-A^+^ cells was significantly decreased after NO training.

However, there was a slight increase of IL-17A^+^ cells after HO training. Thus, additional hypoxia seems to diminish this favorable effect of training. This finding is parallel to the study of Dang et al. reporting that HIF-1 regulates the Treg/Th17 balance in mice, leading to the activation of IL-17 genes and proteosomal degradation of FoxP3 (63). Although we did not measure HIF-1 expression, its induction in the HO group might have contributed to our findings.

Since physical training has been shown to decrease plasma levels of IFN-γ in MS patients (20, 21), we examined the IFN-γ-expression of CD4^+^-enriched cells. However, we found no decrease in the frequency of IFN-γ-producing CD4^+^ cells or in their MFI after our training regimens. Of note, MFI might not always reflect the secreted amount of cytokines with 100% accuracy (64). Thus, further experiments are necessary to validate IFN-γ-expression after training in MS patients.

Although groups in this study were well matched and our training regimen produced considerable effects, HO training did not increase EPO levels and had no additional advantages. This might have been different with higher altitude, training intensity and duration. Overall, a training period of 4 weeks is quite short. A longer training period might have increased VO_2*max*_ and other outcome measures even further. Energy metabolism had to be tested on a bicycle instead of a treadmill, which might have masked training effects. Moreover, longer-term studies could evaluate cognitive decline outcomes like Symbol Digit Modalities Test or Paced Auditory Serial Addition Test (65). However, our training regimen was feasible, well tolerated by all participants and we did not observe any adverse effects of hypoxia. We feel that this positive experience with physical activity was crucial for most participants who increased their activity after the study. Also, immunological data did not indicate that an increased exposure to hypoxia would be favorable.

We investigated metabolic, mental and immunological effects of a moderate treadmill training. We found that both training regimens had considerable but partly differential effects. We assume that hypoxic and normoxic training worked through an improved energy metabolism and immune status, respectively. Both improvements could in turn enhance mood and mobility. Our study strengthens the notion that a moderate and feasible training regimen positively influences factors relevant for the disease course of MS. However, longer-term studies are needed to effectively improve more outcome measures of physical and mental health. Moreover, other immunological parameters implicated with exercise and MS should be investigated in future studies.

## Data Availability Statement

The raw data supporting the conclusions of this manuscript will be made available by the authors, without undue reservation, to any qualified researcher.

## Author Contributions

AM, AB, JS, MK, DM, MB, and FP conceived and designed the study. AM, AB, LK, IC, and US acquired data. AM, AB, LK, IC, JS, MK, and US analyzed and interpreted data. AM and AB wrote the paper. AM, FP, and MB had primary responsibility for final content. All authors contributed to manuscript revision, read and approved the submitted version.

### Conflict of Interest Statement

The authors declare that the research was conducted in the absence of any commercial or financial relationships that could be construed as a potential conflict of interest.
