# Supplementary material for: Metabolic, Mental and Immunological Effects of Normoxic and Hypoxic Training in Multiple Sclerosis Patients: A Pilot Study
Source: Front Immunol. 2018 Nov 29;9:2819. doi: 10.3389/fimmu.2018.02819 (PMC6281996; doi:10.3389/fimmu.2018.02819)
Supplement: Supplementary file 1 [file Data_Sheet_1.pdf]

A

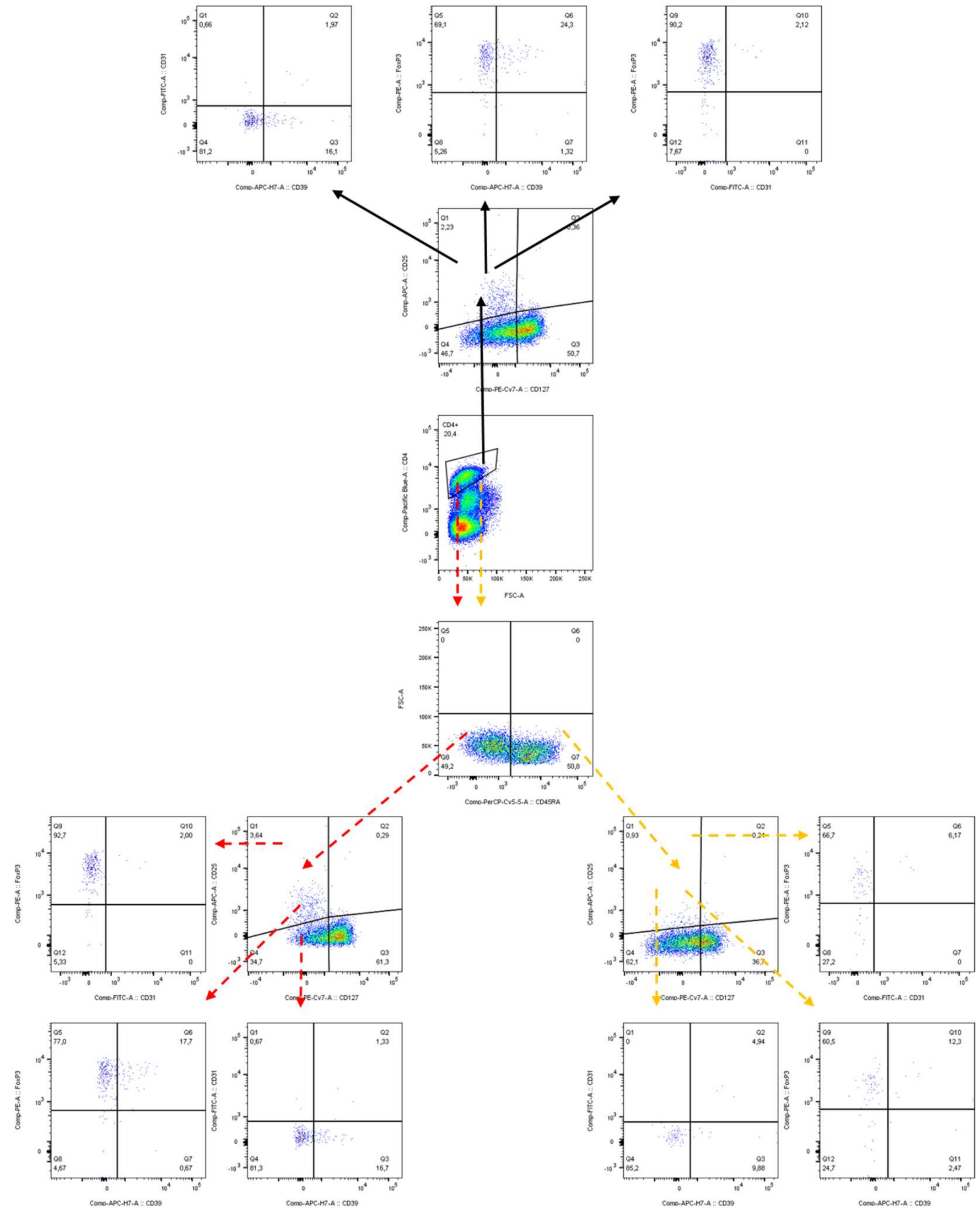

B

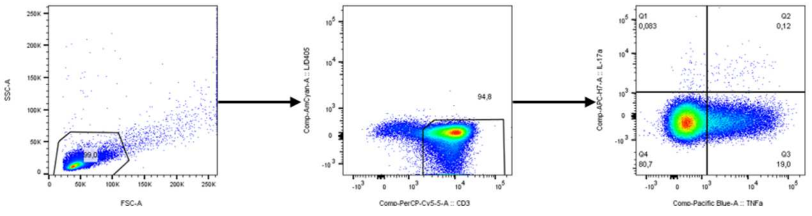

Suppl. figure 1. Gating strategies of **(A)** Treg populations and **(B)** TNFα and IL-17A producing cells

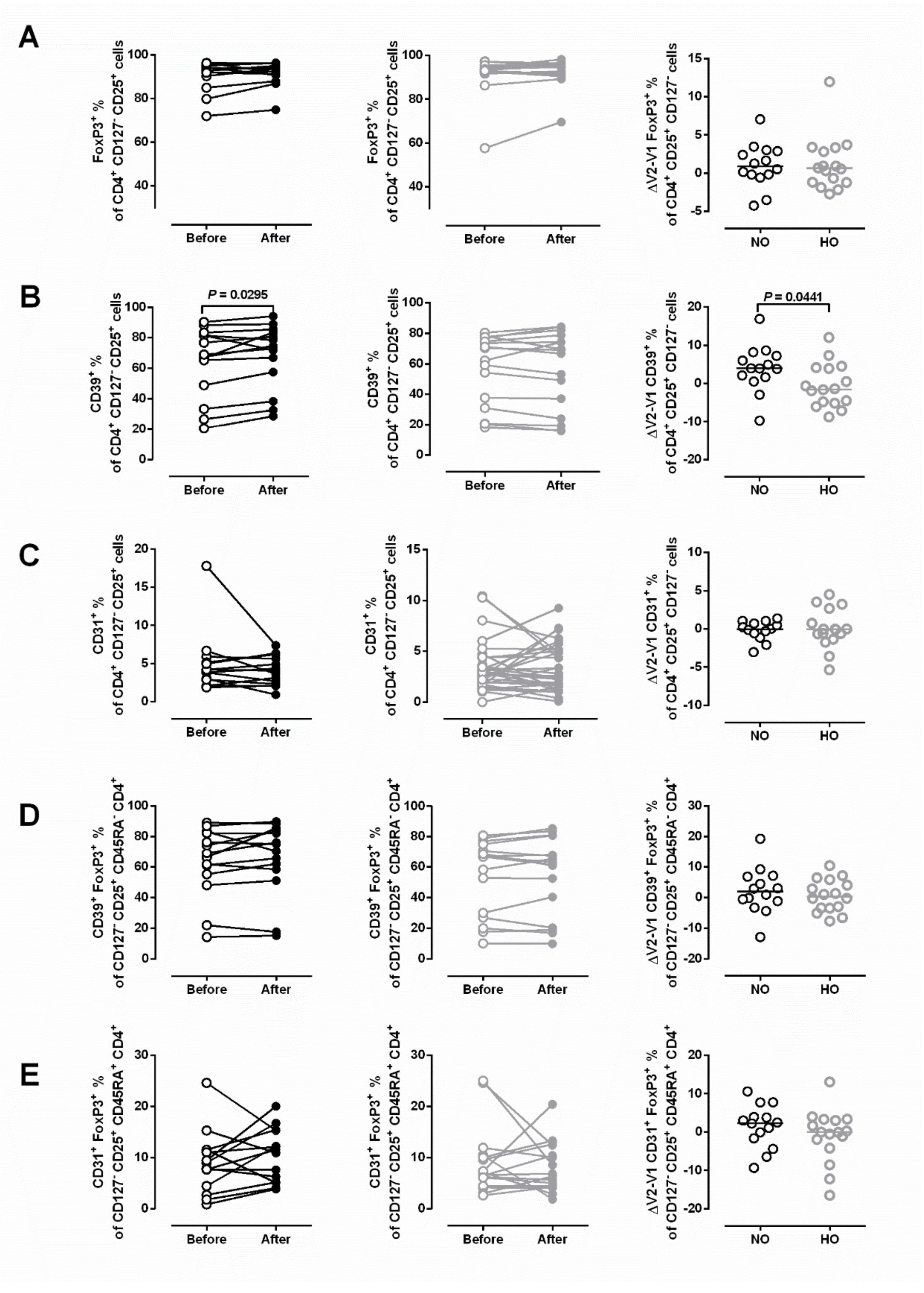

Suppl. figure 2. Frequencies of different Treg populations

(**A**) FoxP3$^+$, **(B)** CD39$^+$ and **(C)** CD31$^+$ of CD4$^+$ CD127$^-$ CD25$^+$ cells. **(D)** CD39$^+$ FoxP3$^+$ of CD127$^-$ CD25$^+$ CD45RA$^-$ CD4$^+$ cells. **(E)** CD31$^+$ FoxP3$^+$ of CD127$^-$ CD25$^+$ CD45RA$^+$ CD4$^+$ cells. All in MS patients after 4 weeks of normoxic (NO, n = 16, black circles) and hypoxic (HO, n = 14, grey circles) treadmill training. Three samples not analyzed due to quality issues. Comparison of training effects (ΔV2-V1, right column). *P* values before vs. after training by Student´s paired t test or Wilcoxon matched-pairs signed rank test. *P* values NO vs. HO by Mann-Whitney U test or Student´s t test

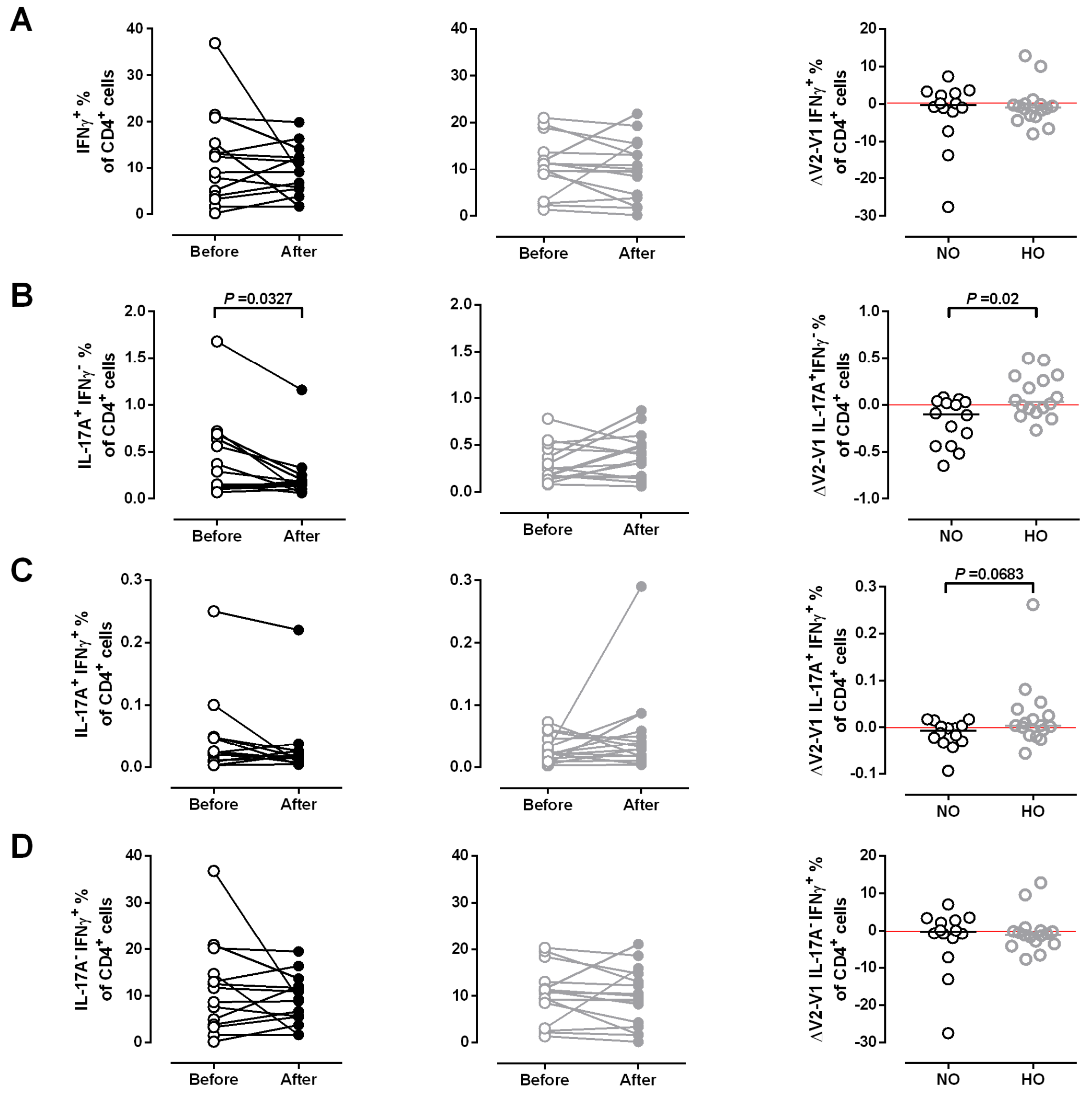

Suppl. figure 3. Frequency of IL-17A and IFN-γ producing T cells

Frequencies of **(A)** total IFN-γ^+, **(B)** IL-17A$^+$ IFN-γ, **(C)** IL-17A$^+$ IFN-γ^+ and **(D)** IL-17A$^-$ IFN-γ^+ of CD4$^+$ cells in MS patients after 4 weeks of normoxic (NO, n = 16, black circles) and hypoxic (HO, n = 14, grey circles) treadmill training. Three samples not analyzed due to quality issues. Comparison of training effects (ΔV2-V1, right column). *P* values before vs. after training by Student´s paired t test or Wilcoxon matched-pairs signed rank test. *P* values NO vs. HO by Mann-Whitney U test or Student´s t test

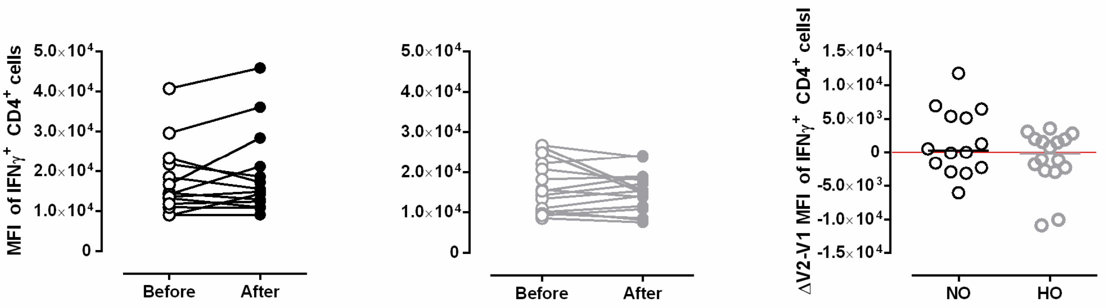

Suppl. figure 4. Median fluorescence intensity of IFN-γ-producing CD4+ T cells

Median fluorescence intensity (MFI) corresponding to the IFN-γ-labelling of CD4+ cells in MS patients after 4 weeks of normoxic (NO, n = 16, black circles) and hypoxic (HO, n = 14, grey circles) treadmill training. Three samples not analyzed due to quality issues
